# An ethnobotanical study in Midyat (Turkey), a city on the silk road where cultures meet

**DOI:** 10.1186/s13002-017-0201-8

**Published:** 2018-02-07

**Authors:** Ali Akgul, Ayfer Akgul, Serdar G. Senol, Hasan Yildirim, Ozcan Secmen, Yunus Dogan

**Affiliations:** 1 Department of Infectious Diseases & Pathology, College of Veterinary Medicine, University of Florida, Gainesville, FL USA; 2 Department of Sustainable Bioproducts, College of Forest Resources, Mississippi State University, Starkville, MS USA; 3 Garden-Herbarium Research and Application Center, Ege University, Izmir, Turkey; 4 Biology Department, Science Faculty, Ege University, Izmir, Turkey; 5 Buca Faculty of Education, Dokuz Eylul University, Izmir, Turkey

**Keywords:** Ethnobotany, Midyat, Medicinal plant, Turkey, Useful plants

## Abstract

**Background:**

Studies of ethnobotanical usages in south-eastern Turkey are rare. To widen this field of knowledge, we conducted an ethnobotanical study in Midyat (Mardin Province), Turkey.

**Methods:**

The field study was completed during three years (2007–2010). Our aim was to document the ethnobotanical uses of local plants and to make an ethnobotanical inventory of uncommon plants using qualitative interviews.

**Results:**

During field studies, 368 voucher specimens were collected in the investigated area. Ninety-two traditionally used plant species were reported from Midyat and surrounding vicinities in Turkey. Among the 92 taxa (129 usages), 35% were used for medical purposes, 22% for food, 13% for animal fodder, 7% as ornamental plants and dyes, 6% as brooms, 4% for latex and as fragrance, 4% for herbal tea, molasses and wine preparation, 3% for agricultural purposes, and 6% for other purposes. Comparative assessment showed that *Teucrium polium* (0.51), *Matricaria aurea* (0.26), *Alcea setosa* (0.21), and *Malva neglecta* (0.21) have the highest recorded UVs_,_ and the following taxa had UVs between 0.10–0.20: *Anthemis cotula* (0.12), *Allium cepa* (0.13), *Alcea striata* subsp. *striata* (0.14), *Crupina crupinastrum* (0.12), *Papaver rhoeas* (0.13), *Salvia multicaulis* (0.14), *Thymbra spicata* (0.11), and *Vicia pannonica subsp. striata* (0.15). We reported the ethnobotanical usages of 21 taxa for the first time, in addition to indicating usages previously recorded in the literature. We also recorded four endemic plant usages in the area: *Alkanna trichophila* var. *mardinensis*, *Centaurea kurdica*, *Centaurea stapfiana*, and *Sideritis libanotica* subsp. *linearis*. They have variable leaf and flower morphology that are used traditionally. They are present as well-developed populations and thus their conservation status is not compromised. Additionally, *Thymbra sintenisii* is a recorded species that is classified as a rare and extensively used species in the region.

**Conclusions:**

These results contribute to the fundamental knowledge of ethnobotanical usages in Midyat. To date, ethnobotanical studies have not been carried out in this region. This investigation uncovered usages of endemic medicinal plant species and traditional knowledge of Midyat communities living in a mixed culture. The people of Midyat, Batman, and Şırnak are Turkish citizens from various ethnic backgrounds, such as Kurdish, Arabic, and Syriac. We compared our data with results from other studies conducted in Turkey, particularly in south-eastern and eastern regions, as well as with studies from bordering countries, Iraq, Jordan, Syria, and Iran. Nonetheless, more work needs to be conducted to extend the present knowledge for locals to contribute to and evaluate economic potential in the region.

## Background

Traditional knowledge of plants has always been transferred from generation to generation throughout the natural course of everyday life [1]. This important knowledge, collated through ethnobotanical studies, is valuable for conservation, and establishment of the local and indigenous plant usages has significant benefits [2]. Turkey has an enormous potential for the exploration of new ethnobotanical usages because of its extremely rich plant diversity [3]. The Turkish flora comprises more than 11,700 plant taxa (about 30% of these are endemic taxa) [4], and the Turkish people have a broad knowledge of folkloric medicines; therefore, Turkey represents a huge resource for ethnobotanical usage studies [5]. Plants are commonly used by Turkish people for traditional remedies, as an herbal tea, food, spice, firewood, dye, furniture, agricultural tools, construction materials, and indoor plants [4]. Many ethnobotanical studies include general medical usage, but these studies are fewer in number than those about non-medical ethnobotanical usages [6].

Recently, botanists in Turkey have started using a different approach in ethnobotanical studies. When they conduct a floristic study in a specific area, an ethnobotanical survey study section is added [7]. Previous studies in Turkey have included a section of ethnobotanical usages at the end of the flora section.

Ethnobotanical studies have been on the increase in many regions of Turkey [1, 8–13]. In Midyat (Mardin Province, Turkey), people benefit from the diversity of flora by using plants as a rich source of medicine. Medicinal plants were used by Anatolian cultures, hence the accumulation of large amounts of remarkable medicinal folk knowledge in the region [14]. Although there are some studies in eastern Anatolia [15, 16], the southeast region of Anatolia is still a poor area in terms of ethnobotany studies. Midyat has a great diversity of plant species given its climatic variation and different ecological habitats. The different ways of life and rich culture in the districts of Midyat have created diverse ethnobotanical usages. One of the oldest traditional plant usages is medicinal, which depends on knowledge and practical experience of using these natural materials. As far as the authors know, this is the first ethnobotanical study conducted in Midyat, Turkey. The primary objective of this study was to identify and document the medicinal ethnobotanical plants and associated ethnobotanical knowledge of the local people. The secondary purpose was to uncover new ethnobotanical usages such as endemic and endangered plant usages, and to evaluate the plant usages in the different ethnic groups in the region.

## Methods

### Study area

**Fig. 1:**
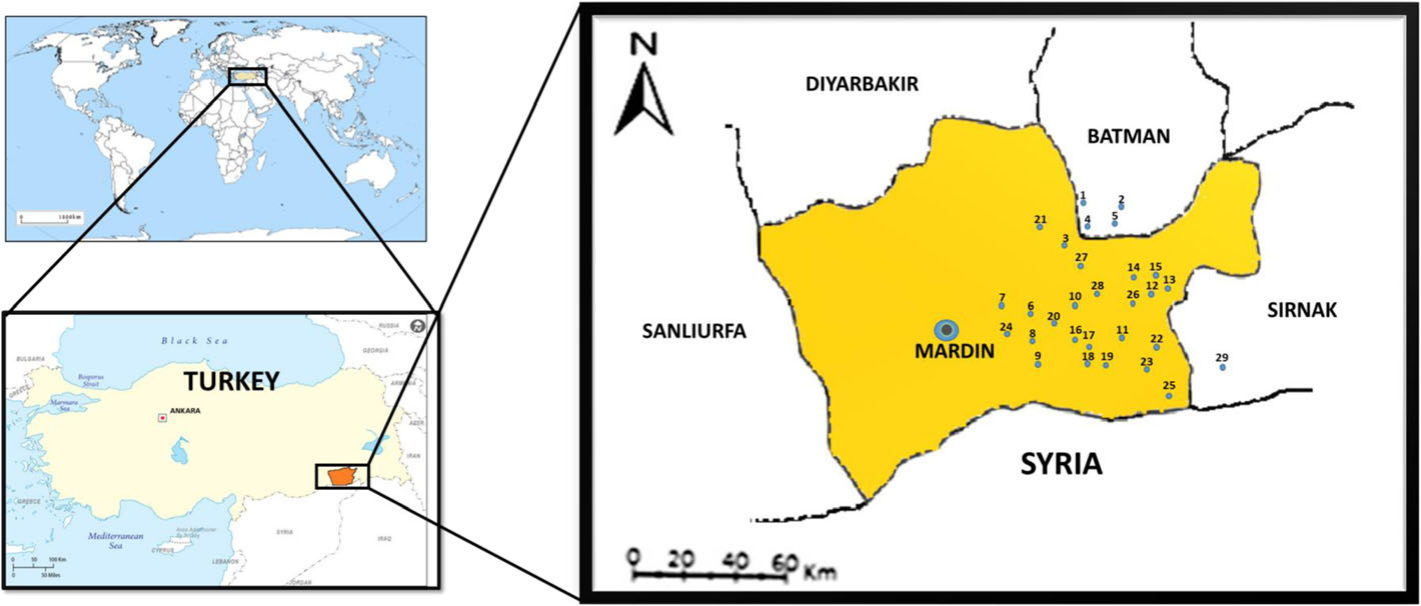
Map of Midyat and districts. Provinces: 1-Doruk, 2-Tasci, 3-Sarikaya, 4-Tepecik, 5-Kayapinar, 6-Ziyaret, 7-Alicli, 8-Harmanli, 9-Camyurt, 10-Dogancay, 11-Yenice, 12-Baglarbasi, 13-Narli, 14-Baristepe, 15-Gulveren, 16-Yolbasi, 17-Pelitli, 18-Yemisli, 19-Sivrice, 20-Budakli, 21-Icoren, 22-Turgali, 23-Eglence, 24-Tepeli, 25-Yayvantepe, 26-Altintepe, 27-Senkoy, 28-Acirli, 29-Haberli

This study was conducted in Midyat, located at 37°25′ *N* – 41°22′ E, in south-eastern Turkey (Fig. 1). The district is located in the south-eastern part of Anatolia. Midyat covers an area of 1394 km^2^ and is located at 1070 m above sea level. The region is rugged and not surrounded by high mountains [17]. The population is 105,952 (2015). In Midyat, there is not only a mixture of cultures, but also a mixture of religions: Syriacs (Christian), Yexidians and Muslims have lived together for thousands of years. Four different languages, Turkish, Arabic, Syriac, and Kurdish, are spoken in this region. For example, in Eglence, a village in the region, people speak Arabic and Kurdish in their daily life. In a Christian village, Haberli, local people speak Arabic, Syriac, and Kurdish. Additionally, in all locations Turkish is the official language; therefore, all young generations speak Turkish very well.

### Socio-economic profile

In Midyat, agriculture, husbandry, and handcrafts are the most important sources of income for the region’s economy. 66.8% of income in the area is from husbandry and agriculture [18]. Traditional hand-made silver products and hand-crafted stonework, fabric painting, and jewelry-making are still important [17]. The most important crops are wheat and barley, followed by cotton, pistachio, olives, grapes, apples, pears, plums, and walnuts [18].

### Ethnographic background

Midyat, formerly known as Matiat, was built in the ninth century BCE by Syriac settlers, and a record of it was found written on Assyrian tablets [17]. The Silk Road is an historic route for overland travelers. The town of Mardin in south-eastern Turkey is an attraction of the Silk Road [19]. The Silk Road is more than just a trade route linking Asia and Europe; it is a display of cultures, ethnicities and religions that have settled in the region, and presents 2000 years of historical and cultural wealth [20]. From east to west, it was used in transporting silk, porcelain, paper, spices, and jewels for cultural exchange between continents [19].

During the Middle Ages, the Silk Road extended along multiple routes from Asia to Anatolia and from Thrace to Europe [21]. The Silk Road in the south of Anatolia passes by Mardin, Adıyaman, Kayseri, Konya, Denizli, and Antalya [19]. Mardin was also an important stopover point along the Silk Road due to its strategic position at the junction of two transit roads [17]. Before the Ottoman empire in the eleventh century, the Seljuk empire – a medieval Turko-Persian-Sunni Muslim empire – provided security by building caravanserais on these roads: there are five inns and caravanserais in Midyat in order to keep the commercial activities in Anatolia alive [19].

### Climate and topography

Midyat is one of the most important floristic regions in south-eastern Turkey. The studied area has a potential to reflect the endemic characteristics of the Irano-Turanian flora and some xeric Indo-Malaysia forms. Summer is very hot, at an average of around 35 °C in June, and winter is cold, with an average temperature of 0.5 °C in January [22].

The study was carried out from 2007–2010, March through late December, when plants were in flowering and fruiting periods. The information on the local names of plants, their usage and preparation were obtained from local citizens (123 respondents) through individual interviews. Most respondents were also asked about the source of their knowledge in order to eliminate information of secondary nature. This information was checked with that from other areas and neighboring villages in order to verify its accuracy. The plants were collected with the help of respondents. In the study area, different religions, languages, and ethnicities are present, which presented us with the advantage of being able to find new and preserved ethnobotanical knowledge from different cultures.

### Ethnobotanical data collection

This study was conducted in Midyat to evaluate the usage of the natural plant flora. Information was gathered by interviewing 123 local people from 30 districts in Midyat and surrounding areas. People showed us plants from the field or dried samples from their properties for our records.

Identification of the specimens from our field collections revealed 92 taxa belonging to 32 plant families. These specimens were identified using the “Flora of Turkey” [23]; we compared them with the specimens in the Herbarium of Ege University, Izmir, Turkey, and listed the names of plant families in alphabetic order. The voucher specimens were also deposited in the Herbarium of Ege University. Plants were identified according to the International Plant Name Index (IPNI: http://www.theplantlist.org/). Plant usages were examined by focusing on natural plant usages, not only on agricultural usages.

Ethno usages of the plants are given under their taxa names with voucher specimen numbers, in alphabetical order. In respective columns, the family, local name, preparation method, and used parts of the plants are recorded. The last column shows literature reports with references (Table 1).

**Table 1:** Ethnobotanical usages of the plants in the study area

Botanical name, specimen number	Family	Local name	Plant part used	Preparation	Use	UV	Reported literature uses
*Alcea setosa* (Boiss.) Alef. A-183	Malvaceae	Hıtmıye (A), hiro (K)	Flower, fruit, root	Infusion, decoction, fruit and root crushed	Cough and flu cure, hair dye, wound healing, labor pain	0.21	Expectorant, diuretic, emollient, edible [31, 35]
*Alcea striata* subsp. *striata* (DC.) Alef. A-50	Malvaceae	Hıtmıye (A)	Flower, fruit, root	Root applied to the skin, infusion	Cough and flu cure, wound healing	0.14	Respiratory disease [15, 54]
*Alkanna trichophila* var. *mardinensis* Hub.-Mor. ^a^ A-91	Boraginaceae	Dıbbeyk (A), Mısmısa (K)	Flower	Latex	Food	0.04	Food [55]
*Allium cepa* L. A-295	Amaryllidaceae	Basal (A)	Leaf	Decoction	Dye for Easter egg	0.13	Dental infections, dye [31, 56, 57]
*Anchusa azurea* Mill. A-85	Boraginaceae	Ivveyne (K), hımhım (A)	Root, aerial parts	Decoction, root crushed for wound	Baskets, wound healing, cancer cure	0.09	Antitumoral, anti-inflammatory, burns and wounds, fresh eaten antihypertensive, carminative, diabetes disease, digestive, rheumatism, wound healing, cold, flu, stomach [11, 46, 54, 58, 59]
*Anchusa azurea* var. *kurdica* (Guşul) D.F*.*Chamb. A-13	Boraginaceae	Gurız (K), hımhım (A)	Aerial part	Fresh eaten	Antidote for animals	0.02	Diaphoretic, diuretic, stomachache, rheumatism, wound healing [45, 47, 60]
*Anchusa strigosa* Labill. A-177	Boraginaceae	Hımhım (A)	Aerial part	Decoction	Cancer treatment	0.02	Wound healing [31]
*Anthemis cotula* L. A-88	Compositae	Kahven (A-K-S)	Aerial part	Decoction, infusion	Herbal tea, treatment for stomach aches and flu	0.12	Cold, hair loss, digestive, laxative, bronchitis [1, 36]
*Aristolochia bottae* Jaub. & Spach A-34	Aristolochiaceae	Gayekakahve (K)	Aerial part	Decoction	Haemorrhoid cure	0.02	Diabetic foot syndrome, wound healing [15, 54]
*Astragalus christianus* L. A-146	Leguminosae	Ğısavitılsağlep (A)	Aerial part	Fresh, dried	Fodder	0.03	Not reported
*Astragalus hamosus* L. A-53	Leguminosae	Kopalehalo (K)	Fruit	Fresh eaten	Food	0.02	Fruit decoction, demulcent and laxative, irritation [61, 62]
*Ballota saxatilis* Sieber ex C.Presl. A-164	Lamiaceae	Rihen (A), tamtam (K)	Aerial part	Hangs on the door	Fragrance	0.02	Antimicrobial, antispasmodic and vermifuge [63, 64]
*Capparis spinosa* L. A-167	Capparaceae	Gayakamber (A)	Aerial part	Infusion	Diabetes treatment	0.02	Pickle, food, fodder, infertility rheumatoid arthritis [15, 16, 40]
*Capsella bursa-pastoris* (L.) Medik. A-38	Brassicaceae	Piçok (K)	Fruit	Fresh eaten	Food	0.02	Expectorant, headache, astringent, emmenagogue, hemorrhoids, wounds, diabetes, spice [29, 38, 46, 47, 65, 66]
*Lepidium draba* L. A-55	Brassicaceae	Kınebıne (K), kırmlıbre (A)	Aerial part	Infusion, cooked by boiling	Food, wound healing	0.09	Eczema, sleep disorder, sedative, anorexia, edible as vegetable [11, 30, 38, 67]
*Centaurea regia* Boiss. subsp. *cynarocephala* (Wagenitz) Wagenitz A-133	Compositae	Hıvhıvok (K)	Root	Fresh root eaten	Food	0.02	Not reported
*Centaurea kurdica* Reichardt ^a^ A-174	Compositae	Cızarılcebel (A)	Flower	Infusion	Kidney ailment cure	0.02	Headache, kidney, rheumatism, sedative [38, 46, 60, 68]
*Centaurea rigida* Banks & Sol. A-162	Compositae	Tahliye (A)	Leaf	Infusion	Antidote	0.03	Not reported
*Centaurea stapfiana* (Hand.-Mazz.) Wagenitz ^a^ -161	Compositae	Tumıke (A)	Aerial part	Fresh or dried used	Fodder	0.08	Not reported
*Prunus mahaleb* L. A-93	Rosaceae	Mahlep (A-K-T)	Fruit, leaf, aerial part	Fresh eaten, leaves used on wound	Diabetes treatment, sugar syrup, amulets	0.06	Fruit, cough, strengthening, expectorate, diuretic, inflammation and respiratory system [38, 41]
*Chondrilla juncea* L. A-190	Compositae	Ğılke (A)	Root	Saps of root collected	Chewing gum	0.03	As a broom, kidney stone, abdominal ache, hemorrhaging [34, 38, 45, 69]
*Cichorium intybus* L. A-180	Compositae	Hındıble (K)	Aerial part	Infusion	Food, cooking	0.03	Burn, stomachache, epilepsy, antihypertensive, prostate, kidney stone, abdominal ache, cardiac disease, blood purifier, uterine disease, emollient, diabetes, depurative [15, 16, 26, 29, 38, 41, 64, 70–73]
*Convolvulus arvensis* L. A-114	Convolvulaceae	Lavlavk (K)	Aerial part	Fresh or dried	Fodder	0.03	Food, constipation, laxative, rope [74–85]
*Crataegus azarolus* var. *aronia* L. A-53	Rosaceae	Alıç (T), ğızran (A-K)	Fruit	Fresh eaten	Food	0.05	Not reported
*Crupina crupinastrum* (Moris) Vis. A-212	Compositae	Maknese ahzar (A)	Aerial part	Aerial parts and branches	Broom	0.12	Infection, antiseptic [86, 106]
*Cyclotrichium leucotrichum* (Stept.) Leblebici A-189	Lamiaceae	Hınne (A)	Aerial part	Hung on the door	Fragrance	0.03	Herbal tea [15]
*Cydonia oblonga* Mill. A-70	Rosaceae	Verekılfercel (A)	Leaf	Infusion	Sore throat cure	0.03	Cardiovascular disease, skin, sensory, diarrhea, beauty, constipation [38, 47]
*Ecballium elaterium* (L.) A.Rich A-51	Cucurbitaceae	Tirozia kera (K), terğuzılıhmar (A)	Fruit	1–2 drops of fruit juice	Sinusitis cure	0.06	Sinusitis, spleen disorder [28, 65]
*Echium italicum* L. A-184	Boraginaceae	Hımhım (A)	Aerial part	Fresh or dried	Fodder	0.02	Wound healing, diaphoretic, emollient, diuretic, ulcer treatment, rheumatoid arthritis [4, 80–82]
*Erodium cicutarium* (L.) L’Hér. A-188	Geraniaceae	Derzikpire (K)	Fruit	Fresh eaten	Food	0.05	Hemorrhoids, anti-inflammatory, diuretic, for constipation, haemostatic, urinary and genital disorders [70, 73, 83]
*Eryngium campestre* L. A-67	Apiaceae	Harşef (A-K)	Aerial part	Stem peeled eaten fresh	Food	0.08	Food, intestinal, hepatitis, digestion, muscle pain [16, 28, 34, 41, 84, 85]
*Euphorbia craspedia* Boiss. A-187	Euphorbiaceae	Lığde (A)	Aerial part	Cover grape molasses	Grape molasses	0.07	Not reported
*Galium aparine* L. A-43	Rubiaceae	Ziven (A-K)	Aerial part	Fresh or dried	Fodder	0.04	Not reported
*Geranium tuberosum* L. A-40	Geraniaceae	Cezuğaraban (A)	Aerial part	Fresh or dried	Fodder	0.03	Eaten fresh [15, 86]
*Gladiolus atroviolaceus* Boiss. A-75	Iridaceae	Cezuğarab (A)	Flower	Fresh eaten	Food	0.03	Eaten fresh, stomachache, colds and flu [15, 64, 87]
*Gundelia tournefortii* L. A-136	Compositae	Kerenk (K), arkue (A)	Aerial part	Stem peeled and eaten fresh	Food	0.04	Vitiligo bronchitis, catarrh, cold, diarrhea, gastric pain, kidney stone, mumps, diabetes [32, 35, 45, 60, 64, 88]
*Hypericum triquetrifolium* Willd. A-201	Hypericaceae	Aran (K), ğırsile (A)	Aerial part	Infusion	Diabetes, cardialgia	0.04	Skin, diabetes, diuretic, sedative, healing injures, antiseptic, diuretic [28, 29, 41, 54]
*Iris reticulata* M.Bieb. A-202	Iridaceae	Bırğızzeyl (A)	Flower	Infusion	Food	0.06	Tonsillitis, herbal tea [50]
*Ixiolirion tataricum* (Pall.) Schult. & Schult.f. A-31	Ixioliriaceae	Terğuzılcebel (A), Hıyar (T-K)	Flower	Latex of flower	Food	0.03	Ornament, sucking [15, 35]
*Juniperus oxycedrus* L. A-178	Cupressaceae	Dıfran (K)	Galbula	Infusion	Cough	0.03	Skin disease, eczema, cholesterol, diabetes, kidney stone, wound, body resistance booster, MS disease, cold, cardiac deficiency, analgesic [84, 89–91]
*Lathyrus inconspicuus* L. A-151	Leguminosae	Şokılgalem (A)	Fruit	Fresh eaten	Food	0.02	Not reported
*Buglossoides purpurocaerulea* (L.) I.M.Johnst A-118	Boraginaceae	Mıjmıjok (A)	Aerial part	Fresh or dried	Fodder	0.02	Not reported
*Malva neglecta* Wallr. A-28	Malvaceae	Toluk (T), hıbbes, tıbbayka (A), tabaknunu (K)	Flower, leaf, aerial part	Crushed flower, root and fruit, flower infusion	Sarma as food, stomachache cure, toys, weight loss, labor pain, kidney diseases, diuretic	0.21	Gastric pain, wound healing, food, skin treatment, against kidney disease and abscesses [1, 15, 18, 26, 34, 35, 46, 60, 68, 92]
*Matricaria aurea* (Loefl.) Sch.Bip. A-32	Compositae	Beybunıc (A), gayeka seva, gihake seva (K)	Flower, leaf, aerial part	Infusion, fresh eaten, hung on the wall	Cough and flu cure, stomach ache cure, bronchial cure, fragrance, soda, cardialgia	0.26	Antispasmodic, analgesic, antipyretic, anticough, antinfluenza, antiasthmatic, antiflatulence, stomachache, cold, throat, back pain, sedative, anti-inflammatory [28, 31, 40, 93]
*Medicago radiata* L. A-207	Leguminosae	Nefel (A)	Aerial part	Fresh or dried	Fodder	0.02	Fodder [94]
*Medicago rigidula* var. *submitis* (Boiss.) Ponert A-208	Leguminosae	Nefel (A)	Aerial part	Fresh or dried	Fodder	0.02	Not reported
*Onopordum carduchorum* Bornm. & Beauverd A-144	Compositae	Kifar (A-K)	Aerial part	Stem peeled, fresh eaten	Food	0.02	Hemorrhoids, food, [15, 88]
*Onosma roussaei* DC. A-155	Boraginaceae	Mısmısılhacel (A) basımbar ılğalem (K)	Flower	Latex of flower	Food	0.03	Not reported
*Paliurus spina-christi* Mill. A-285	Rhamnaceae	Mağaylun (K)	Leaf, flower	Wood	Agricultural tool, headache cure	0.04	Diabetes, nephroplegia, kidney stone, diuretic, fuel [9, 50]
*Papaver rhoeas* L. A-112	Papaveraceae	Ceybuhaten (A), şişık (K)	Aerial part	Boiled, fried eaten	Food, fodder	0.13	Sedative, soporific, coughing, antihemorrhagic [66, 74, 89, 95]
*Parietaria judaica* L. A-170	Urticaceae	Rihen (A-K)	Aerial part	Hung in houses	Fragrance		Hemorrhoids, wound [96, 97]
*Paronychia kurdica* Boiss A-159	Caryophyllaceae	Haşişılselulet (A)	Aerial part	Infusion	Wound and gall bladder treatment	0.04	Antiviral, wound [15, 98]
*Peganum harmala* L. A-293	Nitrariaceae	Harmal (A-K)	Flower, seed	Dried seeds hung on the wall	Amulets and ornaments	0.08	Hemorrhoids, prostatitis, birth, stomachache, snake replant, cardiac pain, muscle spasm, food poisoning, rabies, mental-nervous, digestive, respiratory, skin healing, wound, sedative, palpitation, anti-inflammatory [15, 28–32, 35, 44, 99]
*Petroselinum crispum* (Mill.) Fuss A-87	Apiaceae	Maydanoz (T)	Leaf	Fresh	Decoration material for Easter egg	0.07	Diuretic, weight loss, herbal tea [89, 100]
*Pistacia terebinthus subsp. palaestina* (Boiss.) Engler A-185	Anacardiaceae	Bıtım (A-K)	Fruit	Fresh, fruit crushed for soup	Sore throat cure, as soup	0.04	Colds, flu, diuretic, ulcer, food, wound healing, blood pressure [16, 31, 34, 35, 45, 46]
*Polygonum bellardii* All. A-15	Polygonaceae	Şıbırbat (A)	Aerial part	Dried and rolled	Broom	0.04	Kidney stone, bronchitis, eaten fresh [8, 12, 101]
*Populus euphratica* Oliv. A-154	Salicaceae	Kavak (T)	Stem	Stem cut	Constructional material	0.03	Not reported
*Portulaca oleracea* L. A-64	Portulacaceae	Pırpere (A)	Leaf	Fresh leaves fried	Food	0.14	Fresh vegetable in salad, diuretic, gastrointestinal disorders [50, 70, 77, 91, 102]
*Pterocephalus pyrethrifolius* Boiss. et Hohen. A-160	Caprifoliaceae	Şıttayk (A)	Flower	Fresh and dried	Fodder	0.02	Not reported
*Pyrus communis* L. A-56	Rosaceae	Kımmısre (A)	Fruit	Fresh eaten	Food	0.04	Digestive, catarrh, diarrhea, tonic, food [89, 103]
*Quercus brantii* Lindl. A-59	Fagaceae	Ballot (A-K)	Fruit, bark, shoot, branch	Fresh fruit eaten, dried used	Diabetes, fuel, constructional material	0.05	Food and fuel [77, 104]
*Rhus coriaria* L. A-295	Anacardiaceae	Summek (A-K-T)	Fruit	Fresh eaten	Spice	0.05	Spice, mouth sores, diabetes, relaxation [50, 85, 105, 106]
*Rosa foetida* Herrm. A-81	Rosaceae	Menderis (A-K)	Fruit	Infusion	Cardialgia, cancer treatment	0.03	Not reported
*Rubia tenuifolia* subsp. *doniettii* (Griseb.) Ehrend. & Schönb.-Tem. A-113	Rubiaceae	Zelliko (A-K)	Fruit	Fresh fruit used	Toys	0.04	Diuretic, laxative, emmenagogue, abortive [64]
*Rubus sanctus* Schreb. A-68	Rosaceae	Trureşk (A-K)	Root	Fresh eaten	Food	0.02	Eaten fresh, kidney stone, urinary infection, diabetes, stomachache, eczema [64, 107–109]
*Salvia multicaulis* Vahl. A-142	Lamiaceae	Baravine (K), ikoro (S)	Aerial part	Infusion, applied on wounds	Fodder, wound healing, flu and cough cure, labor pain, anti-inflammatory, antidote	0.14	Inflammatory, analgesic, treatment of stomach disturbances, herbal tea [16, 38, 54, 64]
*Salvia syriaca* L. A-148	Lamiaceae	Sımsım (A)	Fruit	Dried	Spice	0.05	Cough, digestive, flu [22, 100]
*Scabiosa argentea* L. A-213	Caprifoliaceae	Maknese safra (A)	Aerial part	Cut and combined	Broom	0.08	Broom, diuretic, wound healing [15, 45, 54, 76, 110]
*Scandix stellata* Banks & Sol. A-44	Apiaceae	Bızırbenc (A), hıfraf (A-K), ziçırk (K)	Aerial part	Boiled and cooked in oil	Food	0.10	Not reported
*Scorzonera psychrophila* Boiss. & Hausskn. ex Boiss. & Hausskn. A-84	Compositae	Kahfor (A-K)	Root	Fresh eaten	Food	0.03	Not reported
*Scrophularia striata* Boiss. A-158	Scrophulariaceae	Nıkledike (A)	Aerial part	Cut and combined	Broom	0.03	Not reported
*Sideritis libanotica* subsp. *linearis* (Benth.) Bornm. ^a^ A-191	Lamiaceae	Şıltık (K)	Aerial part	Decoction	Herbal tea, broom	0.05	Herbal tea [8, 47, 109]
*Sisymbrium altissimum* L. A-92	Brassicaceae	Şevilgavser (K), hardal (T)	Aerial part	Fresh eaten	Food	0.07	Food, fodder [91]
*Silene dichotoma* Erh. A-105	Caryophyllaceae	Dağnıkestırıye (K)	Aerial part	Fresh eaten	Food	0.03	Food [91]
*Teucrium polium* L. A-182	Lamiaceae	Cığde (A-K-S)	Aerial part	Infusion, decoction	Stomachache cure	0.51	Antispasmodic, antiflatulence, antidiabetic, kidney stone, gastric and kidney pain, diarrhea, headache, purgative, digestive problem, stomachic, liver disorder, diabete, cold, rheumatism [4, 11, 28, 31, 32, 46, 54, 65, 68, 72]
*Thymbra sintenisii* Bornm. & Azn. A-176	Lamiaceae	Zatar (A-K-S)	Aerial part	Decoction with lemon peel	Stomachache cure, spice, jam, ingredient in bread	0.11	Not reported
*Thymbra spicata* L. A-101	Lamiaceae	Catır (A-K)	Aerial part	Decoction	Herbal tea	0.03	Antiseptic, stomach pain [15, 16, 28, 35]
*Torilis tenella* (Delile) Rchb.f. A-130	Apiaceae	Ziçırk (A)	Aerial part	Fresh and decoction	Food, diuretic	0.02	Diuretic [55]
*Tragopogon porrifolius subsp. longirostris* (Sch.Bip.) Greuter A-135	Compositae	Hivhivok (K)	Aerial part	Fried or used fresh	Fodder	0.02	Food, aphrodisiac [50]
*Tribulus terrestris* L. A-173	Zygophyllaceae	Pıruğacuz (A)	Aerial part	Decoction	Kidney disease cure	0.03	Kidney stone, asthma, cardiac disorder [1, 46]
*Medicago monantha* (C.A.Mey.) Trautv. A-120	Leguminosae	Antako (K)	Aerial part	Hung on the wall	Fragrance	0.02	Fodder [103]
*Triticum aestivum* L. A-294	Poaceae	Hınta (A-K)	Aerial part	Dried used to craft	Basket, table, ornament	0.09	Basket [16, 111]
*Urtica dioica* L. A-22	Urticaceae	Gazgazok (A-K)	Leaf	Decoction	Cancer and tuberculosis treatment, sarma as food	0.02	Cancer, cold, diabete, rheumatism, stomach ache, analgesic, arthritis, digestive, diuretic, genital disorder, hemorrhoid, hepatitis, lipsotrichia, rheumatism, anti-inflammatory, astringent, antitussive, respiratory disorder [11, 18, 46, 68, 73]
*Vaccaria hispanica* (Mill.) Rauschert A-121	Caryophyllaceae	Ziven (A-K)	Aerial part	Dried or fresh	Fodder	0.02	Fodder [91]
*Verbascum lasianthum* Boiss. ex Benth. A-05	Scrophulariaceae	Tumıkıtılcereb (A-K)	Root, aerial part	Root ground with raisins	Hemorrhoids, wound healing		Honey [112]
*Vicia hybrida* L. A-124	Leguminosae	Şokıl, şokıla kıtık (K), çelepen (A)	Aerial part	Dried or fresh	Fodder	0.04	Not reported
*Vicia narbonensis* L. A-83	Leguminosae	Şokıl (K)	Fruit	Dried or fresh	Fodder, food	0.07	Food [15]
*Vicia pannonica subsp. striata* (M.Bieb.) Ponert A-149	Leguminosae	Çelepen, çırpenılfara (A), kelle (K)	Fruit	Fresh	Fodder, food	0.15	Not reported
*Vitex agnus-castus* L. A-166	Lamiaceae	Şırte (A)	Aerial part	Cut and combined	Broom	0.05	Not reported
*Vitis vinifera* L. A-210	Vitaceae	Tri (K), ğınıb (A)	Fruit, aerial part	Woods for fuel, boiled in molasses	Sugar syrup, grape molasses, wine, fuel	0.09	Sarma, cough, blood forming [18, 100]
*Xanthium spinosum* L. A-263	Compositae	Kişar (A)	Aerial part	Decoction	Diuretic	0.02	Kidney pain, gastrointestinal disease, fodder, diaphoretic, sedative, exudative [9, 15, 95]
*Xeranthemum annum* L. A-1	Compositae	Sirtık (K), Maknese zarke (A)	Aerial part	Cut and combined	Broom	0.09	Toy, broom [15, 75]

^a^Endemic, *A* Arabic, *K* Kurdish, S Syriac, T Turkish, UV Use value

### Interviews with local people

Information was collected from locals by free-listed observations and semi-structured interviews of people in public areas (generally in fields, tea houses, mosques, churches, village squares, etc.). Local people talked about the collected plants in the fields; the people of Midyat are extremely generous in helping others whom they know. Especially in Midyat, we tried to obtain information from the oldest local people; however, several issues overshadowed data collection. First, people were scared of the threat of terror, which made it difficult to obtain any information. Second, communication sometimes caused problems because of the different languages that people speak in the region. This study was conducted in Turkish, but in order to reach all different groups, assistance was needed. We could only work with local people who spoke the local languages and who were familiar with the people in the area. By using local guidance, we conducted our interviews with local people without much difficulty. Since one of the authors has relations in the area, we got guidance from 15–20 locals with different backgrounds that spoke the same language as local people. We also used guidance to reach more people who have other ethnic or faith backgrounds, such as Syriac Christians. Similarly, teachers, imams (ministers) of mosques and churches, headmen of the villages, and the members of the security services of the villages assisted us in obtaining information from local people. The International Society of Ethnobiology Code of Ethics was taken into account in interviews [24].

### Demographic characteristics of study participants

We conducted face-to-face interviews and surveyed a variety of different participants. We identified and recorded demographic characteristics. We interviewed 123 persons, of which more than 70% were older than 55 years; the mean respondent age was 64 (Table 2). 66% of the participants were male, while female participants made up 34% of the survey.

**Table 2:** Demographic details of the interviewed informants

Category	Subcategory	% of informants
Gender	Male	34.2
	Female	65.8
Age	20–40	9
	40–60	51
	60 and older	40
Education level	None	35
	Primary	59.5
	Secondary	5.5

The demographical characteristics of individuals are illustrated in Table 2. During the interviews, we consulted at least one person who could speak the local language and had a connection with local people. Since the research area was not a secure region, we also had permission from local police officers.

### Quantitative method for analysis

We conducted our study by use value method [25], a quantitative method that demonstrates the relative importance of locally known taxa. It was calculated according to the following formula: Use value (UV), a quantitative method that demonstrates the relative importance of plant species known locally, was also evaluated according to the following formula [26]:

$$ \mathrm{UVi}=\sum \mathrm{Ui}/\mathrm{Ni} $$

where UVi refers to the use value of a species, Ui to the number of citations per specific plant species, and Ni to the number of informants. A high UV indicates the potential importance of the plant species reported.

## Results and discussion

The focus of the study was to identify wild plants, instead of agricultural plants, with medicinal usages. This study will fill a gap in the knowledge about different usages of endemic and rare plants. In this study, Midyat was selected because of the mixed cultures and the fact that no ethnobotanical or floristic studies had been conducted in this region. We focus on: 1) identifying medicinal plant usages 2) determining new ethnobotanical usages and evaluating our findings in terms of cultural ethnobotany.

**Fig. 2:**
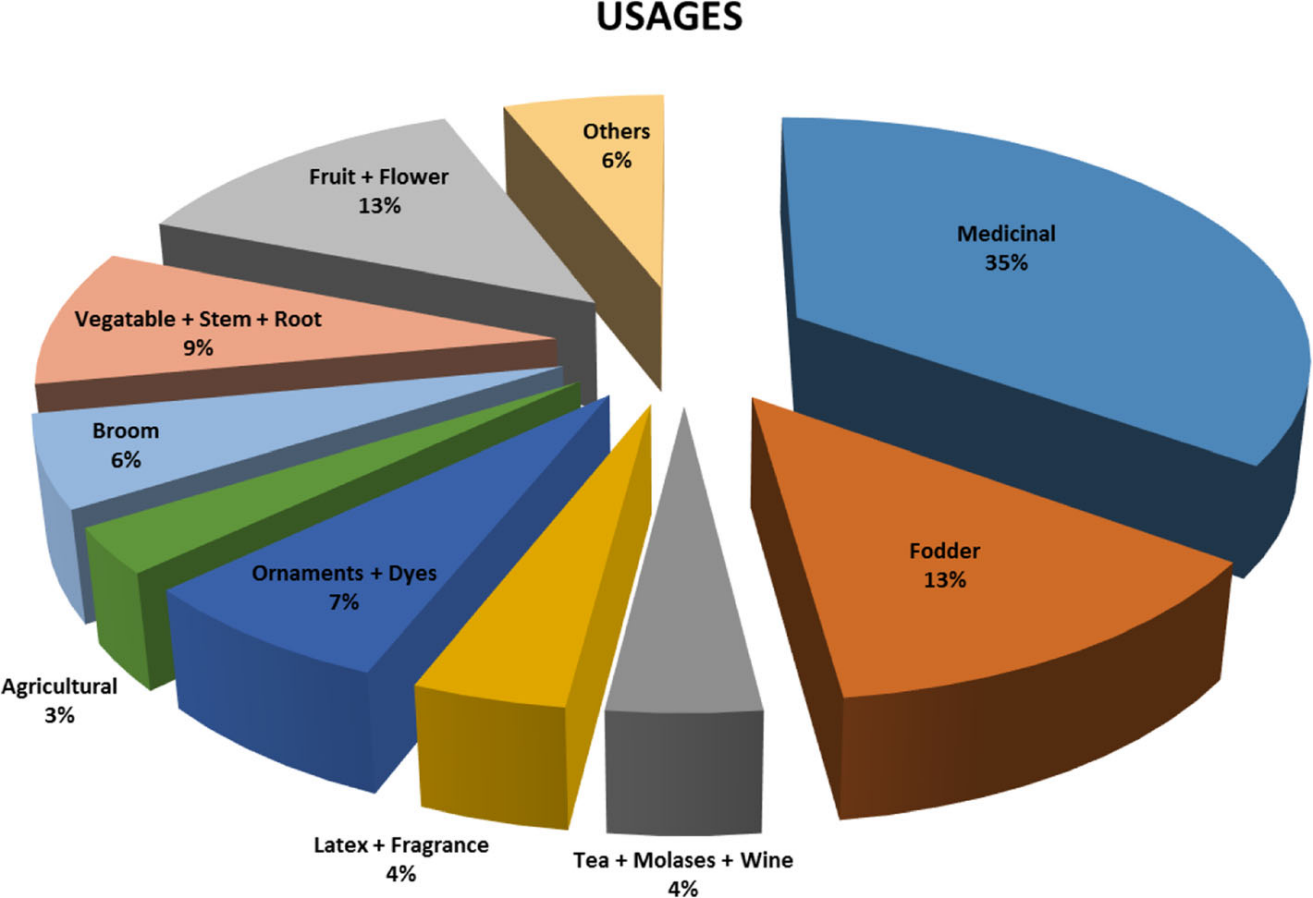
The percentage of ethnobotanical usages in Midyat

In the course of this study, we collected 368 voucher specimens in the investigated area. According to identification results, 92 were traditional plants (81 wild taxa and 11 cultivated plant taxa) (Table 1). Most respondents stated that they had learned the ethnobotanical uses of plants from their parents and elderly relatives. Wild-growing plants were not considered as holding the same economic value as cultivated plants in the studied area. Among the 92 taxa of traditional plants (129 plant usages), 35% were used for medical purposes, 22% for food, 13% for animal fodder, 7% as ornaments and dyes, 6% for broom production, 4% for latex and as fragrance, 4% for herbal tea, molasses and wine preparation, 3% for agricultural purposes, and 6% were used for different purposes (Fig. 2). Within the observed taxa, 21 new ethnobotanical usages were determined in this study.

Local people use various parts of the plants in the region. Of the 92 identified plant taxa, 49% are used for their aerial parts, 20% for their fruits, 9% for their flowers, and 15% for their leaves, stem and roots; in 7%, other parts are used. We compared our findings with previous ethnobotanical studies in Turkey and other countries (Table 1). Our study indicates the importance to document not only medicinal plants, but also edible plants or plants used for fodder, fuel, dyes, and other purposes. In our study, specific and interesting plant usages were determined, such as using the whole of an endemic plant as a broom, herbal tea against cold, or painting Easter eggs with plant dyes. The conservation of this extensive knowledge is crucial, particularly because knowledge is no longer being passed down from older to younger generations.

### Highly utilized species

**Fig. 3:**
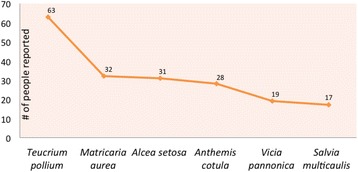
Most common plant usages in Midyat

Use value (UV) is a very important tool for demonstrating the relative importance of medicinal plants in the designated area. Based on our analyses, UV ranges from 0.10 to 0.20 reveal the common ethnobotanical usages for the following taxa: *Anthemis cotula* (0.12), *Allium cepa* (0.13), *Alcea striata* subsp. *striata* (0.14), *Crupina crupinastrum* (0.12), *Papaver rhoeas* (0.13), *Salvia multicaulis* (0.14), *Thymbra spicata* (0.11), and *Vicia pannonica subsp. striata* (0.15). The highest UVs are recorded for *Teucrium polium* (0.51), *Matricaria aurea* (0.26), *Alcea setosa* (0.21), and *Malva neglecta* (0.21) (Fig. 3, Table 3).

**Table 3:** A Important plant usages with preparation and application methods in Midyat 724. B Endemic plants usages in Midyat

Plant scientific name	Aims of plant usage	Preparation and application
**A**		
*Alcea setosa*	Cough and flu	A handful of dry flowers are boiled and drunk during the sickness.
	Hair care dye	The dried fruits are beaten or the flowers applied to wet hair.
	Wound healing	The seeds crushed and applied to the wounds to completely cover it to extract the inflammation.
	Labor pain	A handful of flowers of plant mixed with *Mentha* and boiled.
*Alcea striata* subsp. *striata*	Cough and flu	During flu, the flowers of the plant are boiled and brewed as herbal tea.
	Wound healing	The crushed seeds used to cover the wound area completely in order to extract the inflammation.
*Anthemis cotula*	Stomach pain	Leaves of plant are boiled and used as an herbal tea to treat cold, bronchitis, flu, and stomachache.
	Flu and cold	Herbal tea brewed for cold and flu treatments.
*Crupina crupinastrum*	Broom	Aerial parts and branches used for making broom.
*Malva neglecta*	Food	Fresh aerial parts collected and boiled in water and then fried in oil and mixed with eggs.
	Stomachache	A couple of fresh leaves used in the morning on an empty stomach.
	Toys	Children use their fruit seeds as a toy like marbles.
	Against weakness	When people are critically weak, they have the fresh leaves by adding salt and boiling them. The brew is taken until the patient feels better.
	Labor pain, kidney diseases	Pregnant used by boiling the leaves of the plant. For kidney stones used by boiling the leaves and drinking it like an herbal tea.
*Matricaria aurea*	Cough, flu, bronchial, stomach cure, cardialgia	The plant is boiled and drunk for treatment.
	Food	After the fruit and leaves are washed, they are consumed as a food.
	Fragrance	The whole plants hang on the walls in the entrance of houses to give a good smell.
*Papaver rhoeas*	Food	Fresh young leaves used by boiling and frying for eating. Their flower is added to boiled pounded wheat (bulgur pilav).
	Fodder	For feeding animals; dry and fresh plants used.
*Salvia multicaulis*	Wound healing	After boiling, it is applied to wounds by mixing it with vetch; it is applied at night and kept bandaged until morning.
	Flu and coughs	It is mixed with Imtabso (Papaver) and boiled with some water and is taken.
	Labor pain	The aerial part of plant used as an herbal tea; it is brewed and filtered. People drink it for treatment of labor pain.
	Anti-inflammatory	It is used to treat wounds such as warts and boils; to take out the inflammation, the Plant is boiled and applied on hands at night, kept covered until morning.
	Antidote	It is used when a scorpion bites; plants boiled and the water applied to the bite region and held with bandages.
*Scandix stellata*	Food	The leaves of plant fried and cooked with oil.
*Teucrium polium*	Stomachache	It is brewed for use against stomach pain for children, adults and sometimes for animals.
*Thymbra sintenisii*	Labor pain	Plant used as an herbal tea. It is brewed adding lemon peel and used for labor pain.
	Spice	Used as spice by adding it to salads and dishes.
	Bread preparation	It is also used in preparation of bread; it is mixed with almond and watermelon peel, then fried in olive oil and cooked in a tandoor (oven consisting of a clay-lined large pit).
*Vicia pannonica* subsp. *striata*	Food	The fresh fruit consumed by people by peeling.
	Fodder	For feeding animals whole fruits are used fresh or dried.
*Centaurea rigida*	Antidote	It is used when a scorpion or a snake bites; plant is boiled and the water applied to the bitten region. The treatment continues until healed.
*Euphorbia craspedia*	Molasses preparation	The aerial part of plant is used while boiling the molasses; the plant prevents overflow. The whole plant covers the pan for that purpose.
**B**		
*Alkanna trichophila* var. *mardinensis*	Latex	Latex of flower is used to suck for children.
*Centaurea kurdica*	Kidney disease	The fruit of plant is used after boiling in water; used as a drink.
*Centaurea stapfiana*	Fodder	Dried or fresh aerial part of plant is used for feeding animals intensively in the region.
*Sideritis libanotica* subsp. *linearis*	Herbal tea	The plant is used for brewing and drinking in cold weather.
	Broom	Whole plant used as a broom.

Especially in villages of Midyat, people can easily find highly utilized species. The villagers always keep a dried flowers and plant materials for medical usages or food additives. As an example, *Teucrium polium*, *Matricaria aurea*, and *Alcea setosa* are always presents in many houses in Midyat. Especially, *Teucrium polium* is a well-known remedy in the region, and the dried plant can be found also in local sellers in the city center. Moreover, several other ethnobotanical usages such as soups, ornaments, and fresh plants can be found in local stores in Midyat. Most of plant species that have high UVs can be grown easily such as *Matricaria aurea*, *Alcea setosa,* and *Malva neglecta*. Animal fodders are also common usages in the region because they have grown easily and collected by several villagers in Midyat.

Some highly utilized plant species have already been investigated in terms of biological and chemical properties. For example, *Teucrium polium* has a wide range of applications in the region; owing to unique phytochemicals with new biological activities, more than 130 compounds have been identified so far, and it is used as an anti-inflammatory, antinociceptive, antispasmodic, anticancer, antimutagenic, hypoglycemic, hypolipidemic, hypotensive, anti-ulcer, and antimicrobial [27]. In our study, aerial parts of *Teucrium polium* are used mainly against stomachaches and stomach pains in humans and also animals. Other reported uses of this species are to combat diabetes, diarrhea, and rheumatoid arthritis in Gaziantep [28]; for digestion in Egypt; as food in Albania and Algeria [29]; for liver diseases in Iran [30]; for diabetes and kidney issues in Jordan [31]; and for diarrhea and hemorrhoids in eastern Turkey [32]. Interestingly, the authors were warned that extended use of *Teucrium polium* could cause adverse effects in the liver and kidney [27]. In our study area, there is a very intense usage of *Teucrium polium*, and local people need to be aware of the potential adverse effects of these usages.

*Matricaria aurea*’s essential oils are very rich in chemical compounds, such as phenolic coumarin, which contribute to a moderate antioxidant and antibacterial activity [33]. *Matricaria aurea* has seven different usages: for cough, flu, bronchial complaints, stomachache, cardialgia, as a soda, food, and fragrance. It is mainly used to treat stomachaches and colds by consuming it as an herbal tea. It was reported in previous studies carried out in Jordan that it is used to treat throat and back pains and high blood pressure in children, as a sedative, and an anti-inflammatory substance [31]. *Alcea setosa* (UV 0.21) is traditionally used as a pain reliever, a healing agent, and to treat coughs. *Alcea setosa* is reported for anti-inflammatory treatment of asthma in Jordan with UV 0.11 [31], and the leaves of plant are expectorative and diuretic [14]. The flowers and fruits of *Alcea striata* subsp. *striata* are used as hair dye and in beauty products in Midyat. The same taxon is also used to heal respiratory system disease in Şanlıurfa with a similarly high UV 0.12 [15]. *Malva neglecta* has UV 0.21, and six different usages are reported in Midyat. It is used as a food, against stomachache, for children’s toys, in order to lose weight, against labor pain, kidney diseases, as a diuretic, and to make traditional rolling leaves with rice, called sarma. Several studies reported its use as food [15, 34] and as skin ointment [15, 32]. *Malva neglecta* is used to heal kidney diseases, and in another study from eastern Turkey, the use of this plant to treat abscesses was also reported [35].

In the current study, *Anthemis cotula* is used for several purposes, such as for treating colds by brewing it as an herbal tea, against bronchitis, as a hair treatment, and as a treatment for flu and stomachaches in Midyat. Some similar usages of *Anthemis cotula* are reported for treating colds, hair loss and bronchitis [1, 36]. *Salvia multicaulis* has an interesting usage in the region: as an antidote against scorpion bites (Table 3). *Salvia multicaulis* is also used as an herbal tea in Midyat, Cizre, and in Elazığ [16, 37, 38], and as a spice and to heal skin wounds in the southeast of Turkey [16, 39].

**Fig. 4:**
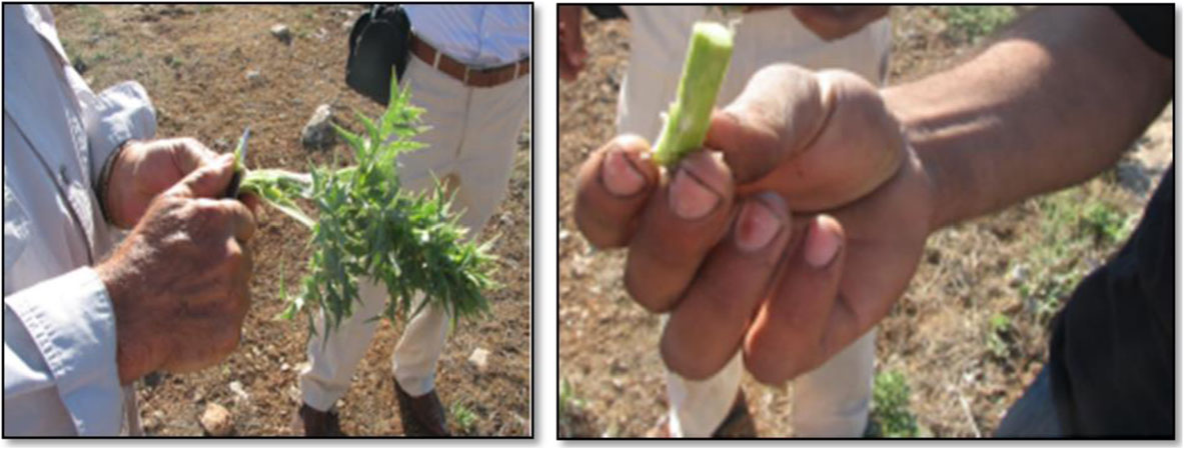
Usage of *Eryngium campestre* by local people in Midyat

**Fig. 5:**
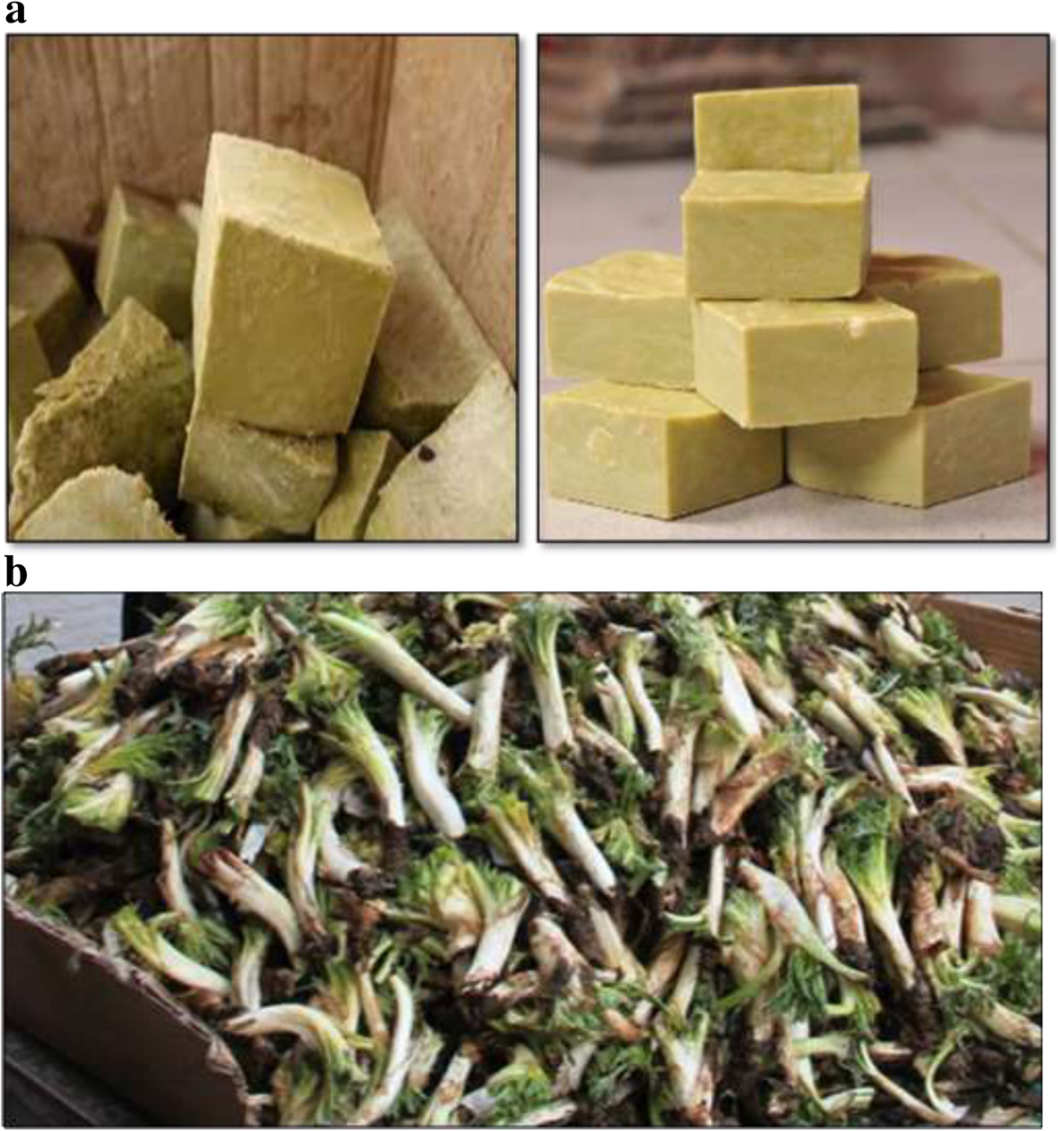
**a.** Bitim soup *Pistacia terebinthus subsp. palaestina* and **b.** *Gundelia tournefortii* sold in local markets

Previously we discussed the ethnobotanical usages with the highest UV value in Midyat. Some less common but no less interesting usages were also reported: *Thymbra spicata* is used in Midyat as an herbal tea for stomachaches. In addition, it is used as an antiseptic [35], as a spice [28], and the leaves of plant are also used in the process of making cheese [37] in southeast regions of Turkey. *Capparis spinosa* is used for diabetes treatment and as an ingredient in pickle. It is used as a pickle, food, and fodder in Şanlıurfa [15, 16], and as a pain reliever and to treat rheumatoid arthritis in Jordan [40]. *Lepidium draba* is consumed as a food in Midyat. In Elazığ province, the fruits of this plant are used as a spice [40], and it is eaten fresh in Iran [30]. Fruits of *Prunus mahaleb* are consumed as a food and against coughing. It is used as a food in Agri [41], and the seeds of plant are used as an expectorative, diuretic substance, and to treat inflammation in Elazığ [38]. *Centaurea kurdica* is boiled in water and used for healing kidney disease. Moreover, the flowers are used as a sedative in Elazığ [38]. *Chondrilla juncea* is used as a gum in Elazığ [38], as a broom [42] and against stomachaches in eastern parts of Turkey [42]. *Eryngium campestre* are peeled and eaten in Midyat (Fig. 4). It is also used to treat intestinal disorders [28], flatulence, hepatitis [41], digestion disorders, and muscle pain [29]. *Eryngium campestre* is consumed as a food in neighboring cities such as Cizre [16]. An unusual ethnobotanical finding was uncovered that children suck the flowers of *Ixiolirion tataricum* in this study area. A similar usage is also reported in Birecik [15]. *Peganum harmala* is known as Syrian rue and Harmal. It has varying pharmacological functions such as analgesic, anti-inflammatory, antioxidant, leukemic, hypoglycemic, and antitumor effects [43]. Nevertheless, its notable usage is for protection against evil spirits in Midyat. The same usage is reported in the southeast of Turkey [15, 28, 35]. It is hung on the wall, especially for the protection of babies against evil spirit. This species is additionally reported for its use against heart disease [28], rabies, snake bites, muscle spasms, and food poisoning [30]. Also, *Peganum harmala* aids in skin and wound healing, as a sedative, to treat inflammations, heart palpitations, nervous system and digestion problems [29, 31]. The seeds of the plant are mixed with honey and used to treat stomach pains [32], and its red fruits used to produce dye in eastern Anatolia [44]. *Pistacia terebinthus subsp. palaestina* has several different usages in the southeast of Turkey. We found that local people make soup with the plant in Midyat (Fig. 5a), and fresh shoots are eaten to treat stomachache. It is similarly used to make soup in neighboring cities [16], and it heals wounds in Malatya [45], where it is also reported as an ulcer treatment [46]. It is an antiseptic and diuretic, used to reduce fever and high blood pressure and to heal ulcers [31]. The young flowers and fruits are consumed against colds and as diuretic agents and its usage as resin, antimicrobial substances and glue have been reported in eastern Turkey [42].

**Fig. 6:**
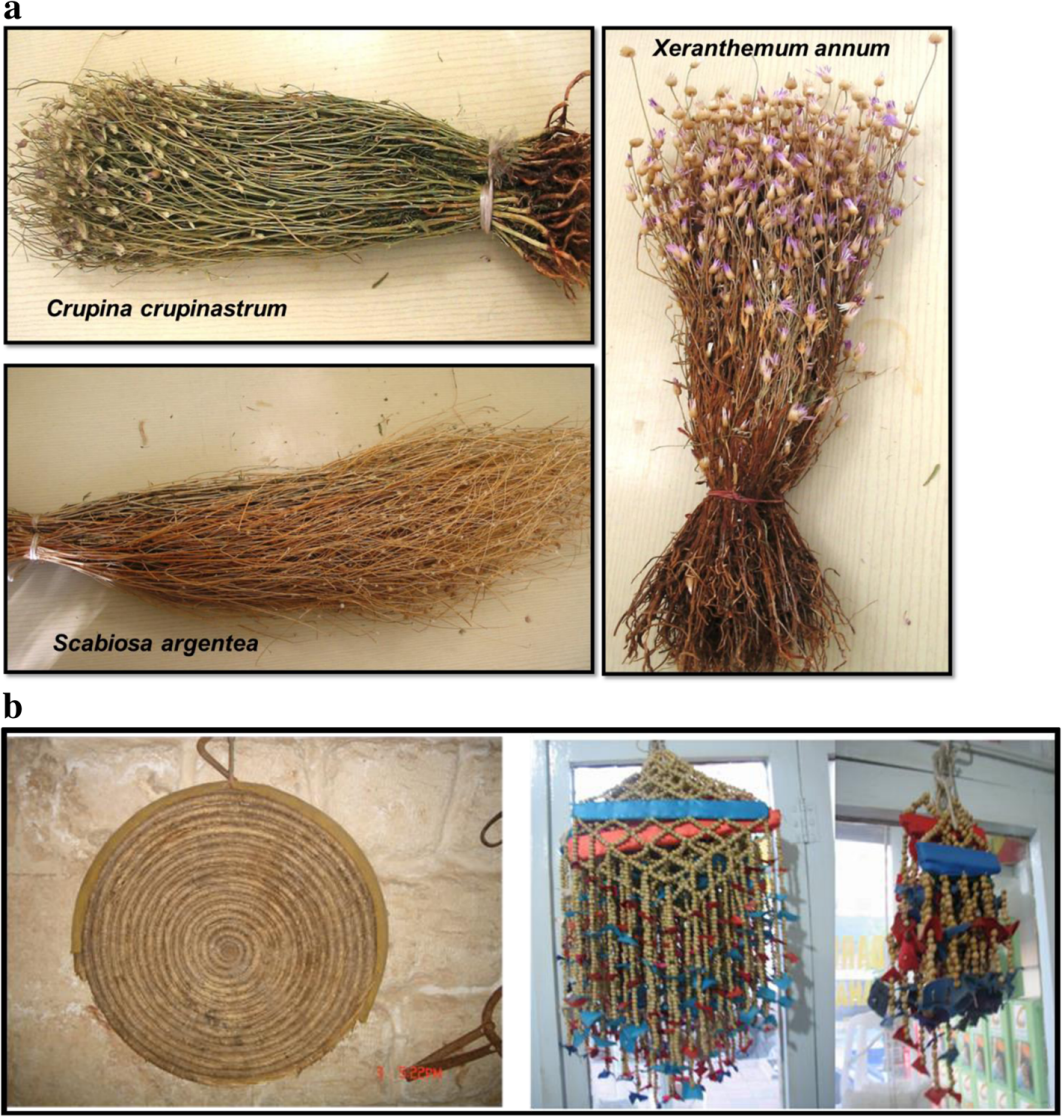
Some interesting usages: **a.** Brooms from Midyat. **b.** Usages of *Peganum harmala* (nazarlik) and *Triticum aestivum* (tray)

In the region, utilizing plants as brooms was a common ethnobotanical usage. *Scabiosa argentea* is used as a broom (Fig. 6a), and the same usage was reported previously in Şanlıurfa and Birecik [15]. Residents of Midyat use *Sideritis libanotica* subsp. *linearis* as a broom and herbal tea. *Sideritis libanotica* was previously also reported as a broom in eastern Turkey [47]. Additionally, *Xeranthemum annum* is used as a broom in Midyat (Fig. 6a). The *X. annum* was similarly used as a broom in Sanliurfa [15]. *Sideritis libanotica* subsp. *linearis* is also used in the region as a broom by using the aerial part of the plant.

**Fig. 7:**
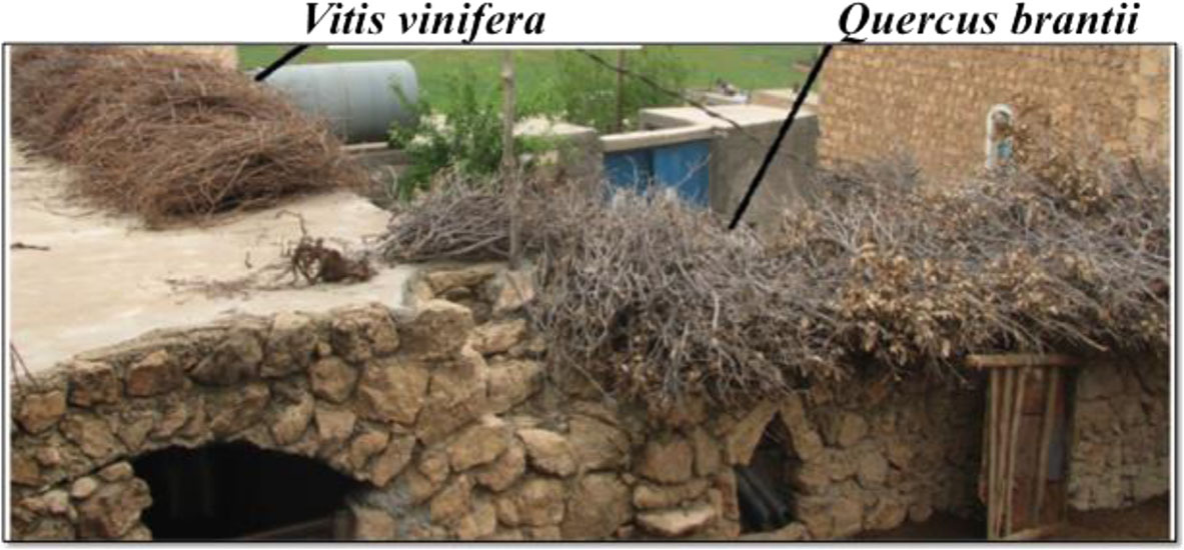
Fuel plants *Vitis vinifera* and *Quercus brantii* stored on the stone houses in Midyat

Some other interesting wood products were found in the region, such as colorful trays and ornaments. As an example, *Triticum aestivum* is used for the production of traditional carrying trays (Fig. 6b). Similar usage of tray-making was determined in Cizre (Şırnak) [16]. *Vitis vinifera* and *Quercus brantii* are commonly used as fuel, and dried branches of these plants are stored on almost all houses in the villages (Fig. 7). People in the region consume many local plants, and sellers use this to their advantage by promoting the locality of the products in Midyat. As an example, stems of *Gundelia tournefortii* are peeled and eaten fresh, and then sold by local people (Fig. 5b).

**Fig. 8:**
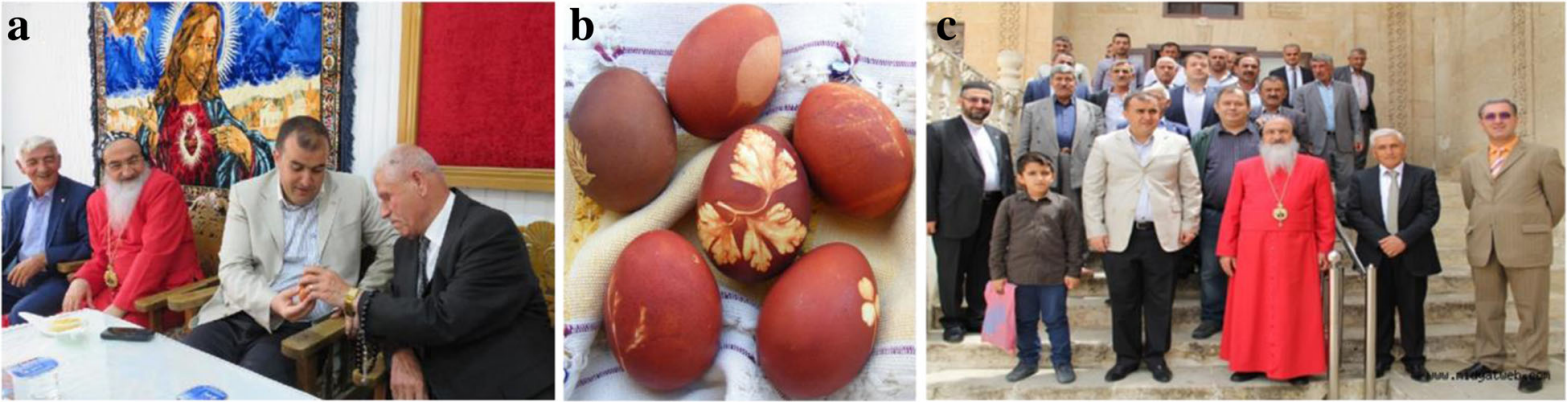
Traditional usages of *Allium cepa* in Midyat. **a.** Government officials celebrate the Easter ceremony by sharing eggs in Midyat [53]. **b.** Dyed Easter eggs by red onion after decorated with parsley leaves. **c.** Local people, religious leaders (pastor and imam) as well as government officials come together in an Easter ceremony by sharing eggs in Midyat [53]. Note: A copyright permission was obtained by copyright holder, Dogus Ofset Gazete ve Matbaacilik

In our study area, perhaps the most picturesque ethnobotanical usage is Easter eggs (Fig. 8a-b). They are prepared at Easter by local Christians called Syriacs. First, wet parsley leaves (*Petroselinum crispum*) were fixed to the egg, and the eggs are boiled slowly with red onion peels (*Allium cepa*) in a pan. After removing the parsley, the Easter eggs are ready (Fig. 8d). In Turkey, you can see some egg painting in large cities such as Izmir. Local sellers sell these eggs, and people like to buy them because of the attractive shapes.

The following plants are used as fragrance at the entrance of houses or on top of front doors: *Ballota saxatilis*, *Cyclotrichium leucotrichum*, *Matricaria aurea*, *Parietaria judaica*, *Trigonella monantha*. Additionally, fruits of *Peganum harmala* and *Prunus mahaleb* are used as ornaments called ‘nazarlık’ against bad spirits, commonly in the southeast of Turkey (Fig. 6b). *Euphorbia craspedia* is locally used to clean the thick bubble layer on grape molasses. Fruits of *Malva neglecta* and *Rubia tenuifolia* subsp. *donietti* are used as children’s toys in Mardin, and the people of Mardin consume the flowers of *Alkanna trichophila* var. *mardinensis*.

Some plants have not previously been reported; these endemic plants are represented in Table 3. Their method of preparation and application are detailed. Four endemic plants were reported (Table 3), *Alkanna trichophila* var*. mardinensis* (Boraginaceae), *Centaurea kurdica* (Asteraceae), *Centaurea stapfiana* (Asteraceae), and *Sideritis libanotica* subsp. *linearis* (Lamiaceae). These plants are used extensively; for example, *Centaurea stapfiana* is used as fodder for animals. This negatively impacts on the conservation of these plants in the region, and there is an issue trying to protect this species as it can only be found in Midyat. Another species, *Centaurea kurdica*, is only traditionally used for healing urinary problems and therefore not as endangered as the previous two species. *Alkanna trichophila* var. *mardinensis* is locally used by children, who consume the flower latex; it is, however, not extensively used and therefore not in danger. *Sideritis libanotica* subsp. *linearis*, whose leaves are used to brew an herbal, and which are also used as brooms, is not a common plant usage, and found in limited locations. The location of *Sideritis libanotica* makes it challenging to collect because it grows in mountains and in shrubs. *Thymbra sintenisii* is used as an herbal tea and spice in Icoren villages. It is a very rare plant and can only be found in two places in Turkey, and construction of highways could badly affect populations of this plant. Even though it is not an endemic species, it has a potential for protection in terms of its pharmaceutical features.

Additionally, we observed many similarities between the names of cultured plants from Midyat and those from bordering countries. These findings show a possible relationship between Jordan and Midyat-Batman in terms of migration and languages. We found similar plant names in Midyat and Jordan for seven species [31]: *Alcea setosa*, *Allium cepa*, *Anchusa strigosa*, *Pistacia terebinthus subsp. palaestina*, *Matricaria aurea*, *Peganum harmala* and *Teucrium polium*. We already know that the common language of the two regions is Arabic. We also compared some of our findings with other studies conducted in bordering countries. For example, Bulgarian–Turk immigrated communities showed more overlapping species among medicinal plants than edible plants in the border regions [48]. In our study, medicinal usages overlapped among different communities. Border regions are important for cross-cultural and cross-border ethnobotany in order to determine changes and variability of medical plant uses and heritage [49]. Even though the region is not very stable right now, an extended study that focuses on cross-border ethnobotany in Turkey, Syria, Iraq, and Iran is needed in future. This is because in the region, only a limited number of ethnobotanical studies have been carried out [15, 16, 50–52].

## Conclusion

Midyat is a city on the Silk Road, which has welcomed very different cultures and religions for thousands of years. The people of Midyat have lived in peace because of their respect and tolerance to others. Even though people in this region have different backgrounds of religion and culture, they have similar botanical knowledge. Other than red peels of *Allium cepa*, which are used for painting Easter eggs*,* they do not have many distinct differences. This type of study will certainly help to uncover, protect, and pass to the next generation the rich cultures of Midyat. The people in Midyat and nearby villages still trust traditional medicine, so in Midyat and its vicinity people continue to use traditional medicines. Our study indicates the importance to document not only medicinal plants, but also edible plants or plants used for fodder, fuel, dyes, and other purposes. We determined the highest UVs for *Teucrium polium* (0.51) and *Matricaria aurea* (0.26); further pharmacological study needs to be done for these species. The conservation of this extensive knowledge is crucial, particularly because knowledge is no longer being passed down from older to younger generations. The use of endemic plants is relatively rare, but *Centaurea stapfiana*, *Thymbra sintenisii* are used extensively, and their conservation status is compromised by their use as food and fodder plants. Additionally, our findings suggested that Midyat and its vicinity might represent a beginning point for further comparative cross-cultural ethnobotany that can contribute to enhancing the current knowledge of folk medicinal plants and lead to conservation plans for protecting rare plant species.

## Abbreviations

UV: Use value
